# Investigating the Impact of Community Volunteerism on the Mental Health of Medical Students

**DOI:** 10.1177/23821205231191903

**Published:** 2023-07-31

**Authors:** Sheina A. Duncan, Gabriel L. Sperling, Matthan I. Moy, Regina Hansen, Chris K. Soudah, Patrick Rodriguez, Andrea Rego, Vivian N. Rojas, Melody Paul, Joseph A. Robicheaux, Carrie Chen, Christopher Thang, Denny Fe G. Agana

**Affiliations:** 1 John Sealy School of Medicine at the University of Texas Medical Branch at Galveston, USA; 2 12340 The University of Texas Health Science Center at Houston, USA; 3 Department of Epidemiology, Director of Diversity, Equity & Inclusion, and Director of the MPH Program School of Public and Population Health at the University of Texas Medical Branch at Galveston, USA

**Keywords:** medical students, community service, perceived stress, mental health, public health

## Abstract

**OBJECTIVE:**

This study aimed to analyze the impact of community service on the mental health of medical students through their perception of stress.

**METHODS:**

The 10-item Perceived Stress Scale was used to measure the stress levels of 82 medical students over a 3-month period. Additional survey questions gauged students’ weekly volunteer experiences in clinical and nonclinical settings and their perceived effects on stress and quality of life.

**RESULTS:**

Results found an inverse relationship between the number of clinical volunteer hours and perceived stress (*P* = .0497). Nonclinical and total volunteer hours were correlated with both reduced perceived stress levels (nonclinical *P* = .0095, total *P* = .0052) and better quality of life (nonclinical *P* = .0301, total *P* = .0136). All individual perceived stress scores fell into the low or moderate stress ranges of the Perceived Stress Scale per the week-to-week analysis.

**CONCLUSION:**

The preliminary results raised important research questions about the impact of volunteering on medical student perceived stress. As medical students face higher levels of stress in comparison to the general population, it is exceedingly important to determine methods to decrease their risk of compromising their mental health. This study may aid in decision-making and research in favor of or against offering community service opportunities as part of the core medical education curriculum.

## Introduction

Prior to entering medical school, students report rates of perceived stress similar to that of the general population. However, studies indicate that being a student in the medical education system negatively impacts mental health, in which medical students report higher levels of psychological distress and suicidal ideation than their age-matched peers.^1^ Decreased mental health over the course of training in medical school suggests this issue is chronic rather than episodic.^2^ Furthermore, the prevalence of burnout in medical student populations is a major concern within medical education and healthcare institutions, creating significant consequences that continue into residency and beyond.^3^ A lack of focus on mental wellness intervention during medical education may have consequences past medical school and result in suboptimal patient care and decreased personal health of future physicians.^4^

The relationship between the amount or severity of stress, perception of stress impacting mental health, and subsequent mental health status is neurologically complex. Individually, medical students adapt various coping strategies to reduce stress and burnout, such as exercise.^5^ Systemically, some medical schools have changed their grading schemes from a numerical and lettering system to a pass–fail grading system to improve student well-being. Though improvements in psychological well-being have been reported, higher-than-normal levels of anxiety, stress, and depression persist.^5^ This result yields further exploration into potential activities that can reduce poor stress outcomes and improve an individual's welfare. One common, relevant activity that is shared among both medical students and the general population is volunteerism in community service.

Research on volunteerism and its impact on health suggests the use of community service may be a possible intervention for favorable outcomes on depression, life satisfaction, and well-being.^6,7^ Yeung and colleagues suggest that volunteering should be promoted by public health officials as a healthy lifestyle due to its positive influence on a variety of mental and physical health outcomes as well as life satisfaction.^8^ The Association of American Medical Colleges’ (AAMC) *Report on Residents* provides annual statistics about the characteristics of former medical students who are now resident physicians.^9^ The number of volunteer experiences is measured, along with research and work experiences.^9^ Thus, most medical students participate in volunteerism, making it an important structure within medical education impacting students. To date, no studies of this nature have been examined in medical student populations, despite a long tradition of medical student volunteerism.

As medical students face higher levels of stress in comparison to the general population, it is exceedingly important to determine methods to decrease the risk of compromising their mental and physical health. Thus, the primary aim of this study was to analyze the impact that service-oriented volunteerism has on the perceived stress of medical students. Examining this more closely can aid in a better understanding of preventative mental healthcare for medical students and support decision-making regarding the inclusion of community service components as part of the core medical education curriculum. A second aim of the study, due to the differences in risk of harm in clinical versus nonclinical settings, evaluated if the type of volunteerism had an impact on postexperience perceived stress. Based on associations investigated in the aforementioned studies, the following two hypotheses were tested regarding correlations between perceived stress and community service:

1. Volunteer experiences decrease postexperience perceived stress levels.
2. Non-clinical volunteer hours decrease postexperience perceived stress and increase quality of life levels compared to clinical volunteer hours.

## Methods

### Participants

Data were collected from 82 medical students who attend a nationally recognized medical school in a federally underserved region of the southwest United States. Participants consisted of first- and second-year medical students who applied to volunteer (self-selected) in a community service organization that tutors and mentors second through eighth-grade students from a local public school district. Students from other local institutions and nonmedical students were excluded from this study analysis. Medical students with treated or undiagnosed mental health concerns were not excluded. APA ethical standards were followed in the conduct of the study. The UTMB Institutional Review Board (IRB) Chairman or designee reviewed this study and determined that this submission does not meet the definition of “human subject research,” as defined by the regulations at 45 CFR 46.102 as this study involves Quality Assessment/Quality Improvement. Therefore the project does not require IRB approval or oversight. Written informed consent was obtained from all subjects for publication of this study.

### Measures

The Perceived Stress Questionnaire (PSQ), shown in Table 1, included the 10-item Perceived Stress Scale (PSS) and 4 additional questions to gauge the students’ weekly volunteer experiences in both clinical and nonclinical settings.^10^ The PSQ utilizes a 5-point Likert scale response, except one additional question was added in this study where students were asked to specify the number of volunteer hours completed in that week.

**Table 1.** Survey implementing perceived stress scale and clinical/nonclinical volunteering questions for medical students.

In the last week, how often have you been upset because of something that happened unexpectedly? (0 = never, 1 = almost never, 2 = sometimes, 3 = fairly often, 4 = very often)
In the last week, how often have you felt that you were unable to control the important things in your life? (0 = never, 1 = almost never, 2 = sometimes, 3 = fairly often, 4 = very often)
In the last week, how often have you felt nervous and “stressed”? (0 = never, 1 = almost never, 2 = sometimes, 3 = fairly often, 4 = very often)
In the last week, how often have you felt confident about your ability to handle your personal problems? (0 = never, 1 = almost never, 2 = sometimes, 3 = fairly often, 4 = very often)
In the last week, how often have you felt that things were going your way? (0 = never, 1 = almost never, 2 = sometimes, 3 = fairly often, 4 = very often)
In the last week, how often have you felt that you could not cope with all the things that you had to do? (0 = never, 1 = almost never, 2 = sometimes, 3 = fairly often, 4 = very often)
In the last week, how often have you been able to control irritations in your life? (0 = never, 1 = almost never, 2 = sometimes, 3 = fairly often, 4 = very often)
In the last week, how often have you found that you were on top of things? (0 = never, 1 = almost never, 2 = sometimes, 3 = fairly often, 4 = very often)
In the last week, how often have you been angered because of things that were outside of your control? (0 = never, 1 = almost never, 2 = sometimes, 3 = fairly often, 4 = very often)
In the last week, how often have you felt difficulties were piling up so high that you could not overcome them? (0 = never, 1 = almost never, 2 = sometimes, 3 = fairly often, 4 = very often)
How many clinical volunteering hours did you complete this past week? (this includes time spent at St. Vincent's, etc)
How many nonclinical volunteering hours did you complete this past week? (this includes time spent mentoring/tutoring with Connect)
On a scale of 1 to 5, how much did your volunteering in the past week affect your stress levels? (1 = reduced my stress a lot, 2 = reduced my stress a little, 3 = no impact, 4 = increased my stress a little, 5 = increased my stress a lot)
On a scale of 1 to 5, how much did your volunteering in the past week improve your quality of life? (1 = reduced my quality of life a lot, 2 = reduced my quality of life a little, 3 = no impact, 4 = increased my quality of life a little, 5 = increased my quality of life a lot).

### Procedures

Students completed PSQs at the start of the program to serve as a baseline and after each of the program's remaining weekly volunteer events in September through December 2021. Each student completing the PSQs was assigned a unique number to track their completed surveys throughout the duration of the program. Additionally, the data were collected from weekly PSQs and input via Google Forms that auto-populated responses into an Excel sheet.

### Statistical analysis

The mean and standard deviation of the responses for each question were measured at baseline and at the end of the program. The differences between the responses at the two time points were assessed using the Wilcoxon signed-rank test. Levels of stress were determined using the sum of the PSQ responses after adjusting for negative questions (low: sum < 13, moderate: 13-26, high sum≥27). The frequencies of students at each level were assessed each week. The correlation between the number of clinical, nonclinical, total volunteering hours, stress, and quality of life was assessed using Spearman's Correlation. The data were analyzed using SAS 9.4 software (SAS Corp, Cary, North Carolina).

## Results

The PSQ Pre and Post results are displayed in Table 2. Question 4 (In the last week, how often have you felt confident about your ability to handle your personal problems?) was statistically significant (*P* = .0020), with a preliminary mean of 0.69 (SD: 0.66) which increased to 1.01 (SD: 0.73) in the postsurvey.

**Table 2.** Perceived stress scale pre and post results of survey.

QUESTION:	BASELINE		POST		*P*-VALUE
	M	SD	M	SD	
1. In the last week, how often have you been upset because of something that happened unexpectedly?	1.05	0.80	1.23	0.80	.10
2. In the last week, how often have you felt that you were unable to control the important things in your life?	1.20	0.93	1.24	1.05	.76
3. In the last week, how often have you felt nervous and “Stressed”?	2.16	0.84	2.00	0.84	.23
4. In the last week, how often have you felt confident about your ability to handle your personal problems?	0.69	0.66	1.01	0.73	.0020
5. In the last week, how often have you felt that things were going your way?	1.15	0.61	1.20	0.62	.25
6. In the last week, how often have you felt that you could not cope with all the things that you had to do?	1.07	0.95	1.17	0.99	.45
7. In the last week, how often have you been able to control irritations in your life?	0.76	0.61	0.88	0.68	.09
8. In the last week, how often have you found that you were on top of things?	1.25	0.68	1.24	0.61	.61
9. In the last week, how often have you been angered because of things that were outside of your control?	1.11	0.81	1.16	0.90	.82
10. In the last week, how often have you felt difficulties were piling up so high that you could not overcome them?	1.04	0.89	1.15	0.98	.49

**Figure 1.**
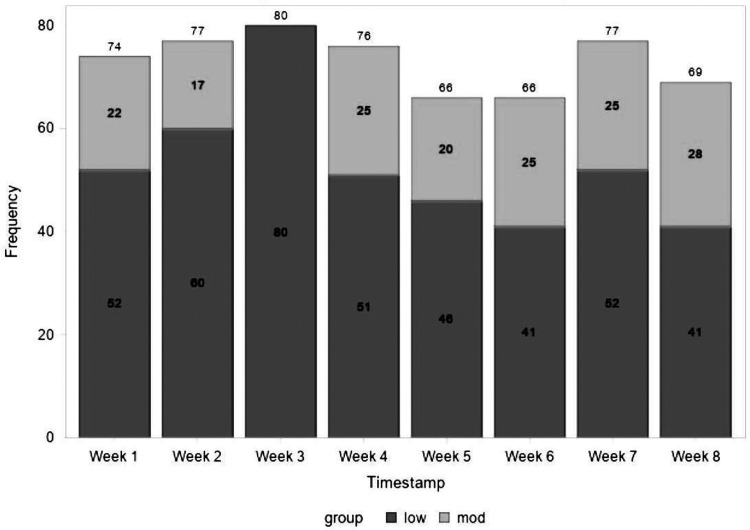
Frequencies of Medical Students in Stress Groups Determined by Perceived Stress Scale Calculation, Collected During September–December 2021.

There were multiple correlations between volunteer hours and the two additional questions regarding stress levels and quality of life (Table 3). The number of clinical volunteering hours the students participated in that week had a positive association with the perceived impact those experiences had on their stress levels (*P* = .0497), indicating that the greater number of clinical volunteer hours participated in, the less stress they perceived. Both nonclinical volunteer hours and total volunteer hours were correlated with both reduced perceived stress levels (nonclinical *P* = .0095, total *P* = .0052) and quality of life (nonclinical *P* = .0301, total *P* = .0136). The week-to-week analysis showed that all individual perceived stress scores fell into the low or moderate stress ranges of the PSS (Figure 1).

**Table 3.** Correlation between volunteering and stress levels and quality of life.

QUESTION	MEAN	SD	STRESS LEVELS 2.37 (0.83)		QUALITY OF LIFE 2.44 (0.90)	
			RHO	*P*-VALUE	RHO	*P*-VALUE
How many clinical volunteering hours did you complete this past week? (this includes time spent at St. Vincent's, etc)	0.78	1.54	−0.08	.0497	−0.05	.21
How many nonclinical volunteering hours did you complete this past week? (this includes the time you just spent mentoring/tutoring with Connect)	1.61	1.52	−0.1	.0095	−0.08	.03
Total hours	2.38	2.27	−0.11	.0052	−0.1	.01
(1 = reduced my stress a lot, 2 = reduced my stress a little, 3 = no impact, 4 = increased my stress a little, 5 = increased my stress a lot).						

## Discussion

Most notably, the data highlights an association between the number of volunteer hours and perceived stress levels. Data shows that higher total volunteer hours are associated with lower perceived stress levels for all volunteer types (*P* = .0052), and that nonclinical volunteering is associated with a better quality of life (*P* = .0301). These observations further support existing literature that links volunteerism with decreased stress levels, particularly nonclinical volunteering having a potentially even larger benefit than clinical volunteering.

Additionally, the unique nature of a mentor-mentee relationship, emphasized in this particular program's curriculum, encourages the cultivation of strong bonds over the duration of the semester. Student-guided programs, such as the one studied here, present strong support and positive strategies for students coping with stress.^11^ Future studies should assess this as a potential explanation for the connection between nonclinical volunteering and changes in perceived quality of life shown in this study to determine whether nonclinical efforts may be more appreciated as an outlet for medical students. Furthermore, studies could assess whether nonclinical experiences may be associated with lower perceived personal risk than that f clinical experiences, as medical students are not managing patient health outcomes during nonclinical experiences like they are during clinical experiences.

Preliminary survey results indicated that the surveyed sample population had low perceived stress levels. Surveying medical students at the start of the semester before many school-induced stressors arise may have been a factor. This may also suggest that medical students operate at a baseline of high stress, which may be perceived as a lack of stress. Additionally, students may perceive low stress while other psychosocial and physiological measures indicate high stress. Stress levels of students may increase as the semester progresses, likely attributed to academic performance, deadlines, sleep deprivation, and financial burdens. For this reason, some might expect to see increases in perceived stress levels from the pre- to post-survey. However, our data indicate otherwise and showed an increase in the students’ self-efficacy throughout the semester while volunteering, as indicated by the results of Question 4 in Table 2. The community service experience may be offsetting the expected increase in perceived stress levels, which may suggest decreased perceived stress levels due to the experience. Other stress management mechanisms, such as exercise or social activities, may have also contributed and were not controlled for in this study. Future studies would need to adjust for physiological stress indicators, as they may affect perceived stress scores. Also, obtaining a physiological and perceived stress analysis prior to the start of medical school classes, to be used as a baseline compared to moments throughout the medical education, may help isolate causes of stress and effective stress management mechanisms.

This study highlights the complexities of assessing stress levels for medical students. There is likely great variation week by week in confounding and moderating factors influencing each individual first- and second-year medical student that were not controlled for in this study. For example, some weeks have more classes, exams, research projects, etc which can impact perceived stress levels. Additionally, there are other personal stressors, such as managing finances, family/relationships, and COVID-19 that were not specifically identified. Most importantly, stress is multifaceted and longitudinal, so it needs to be monitored over time with multiple physiological and psychosocial indicators. Thus, future studies should incorporate additional methods of measuring stress and stress management aside from the PSQ, including an investigation of physiological stress.

Other limitations of this study include volunteer cohort absences and a restricted sample size of medical student volunteers from one academic institution and within a single service program (*n* = 82). Thus, sampling bias may have been present. However, a power analysis for the paired test comparing baseline and post-program suggested the analysis needed 10 pairs for a power of 80%, indicating the sample size was sufficient. Medical students who elect to participate in community service programs may have more time and/or less stress compared to their classmates. In future research, a more representative sample size of medical students from multiple institutions or volunteer settings (clinical and nonclinical) should be gathered to mitigate the risk of introducing bias. Similarly, social desirability bias may have also been contributory during this data collection period, as the students may have given responses they deemed more socially acceptable. In future studies, special consideration and additional measures should be given when asking medical students questions about their mental health.

## Conclusion

As medical students continue to play a key role in healthcare, it is important to understand the factors that impact their perceived stress and mental health. More medical schools have realized the drastic decline in mental health and high stress that medical students experience, which has led to some schools prioritizing mandatory courses in well-being promotion. This study shows that community service components incorporated into the core curriculum may be a plausible solution to ease the mental burden seen in medical education. Medical institutions should consider further investigating this solution.
